# Single-Cell Landscape of Transcriptional Heterogeneity and Cell Fate Decisions during Mouse Early Gastrulation

**DOI:** 10.1016/j.celrep.2017.07.009

**Published:** 2017-08-01

**Authors:** Hisham Mohammed, Irene Hernando-Herraez, Aurora Savino, Antonio Scialdone, Iain Macaulay, Carla Mulas, Tamir Chandra, Thierry Voet, Wendy Dean, Jennifer Nichols, John C. Marioni, Wolf Reik

**Affiliations:** 1 Epigenetics Programme, Babraham Institute, Cambridge CB22 3AT, UK; 2 EMBL-European Bioinformatics Institute (EMBL-EBI), Wellcome Genome Campus, Cambridge CB10 1SD, UK; 3 Wellcome Trust Sanger Institute, Single-Cell Genomics Centre, Cambridge CB10 1SA, UK; 4 Wellcome Trust-Medical Research Council Stem Cell Institute, University of Cambridge, Tennis Court Road, Cambridge CB2 1QR, UK; 5 Department of Physiology, Development and Neuroscience, University of Cambridge, Tennis Court Road, Cambridge CB2 3EG, UK; 6 Department of Human Genetics, Human Genome Laboratory, KU Leuven, 3000 Leuven, Belgium; 7 Cancer Research UK Cambridge Institute, University of Cambridge, Cambridge CB2 ORE, UK; 8 Centre for Trophoblast Research, University of Cambridge, Cambridge CB2 3EG, UK

**Keywords:** gastrulation, embryo, single-cell RNA-seq, epiblast, primitive endoderm, primitive streak, X-chromosome, transcriptional noise

## Abstract

The mouse inner cell mass (ICM) segregates into the epiblast and primitive endoderm (PrE) lineages coincident with implantation of the embryo. The epiblast subsequently undergoes considerable expansion of cell numbers prior to gastrulation. To investigate underlying regulatory principles, we performed systematic single-cell RNA sequencing (seq) of conceptuses from E3.5 to E6.5. The epiblast shows reactivation and subsequent inactivation of the X chromosome, with *Zfp57* expression associated with reactivation and inactivation together with other candidate regulators. At E6.5, the transition from epiblast to primitive streak is linked with decreased expression of polycomb subunits, suggesting a key regulatory role. Notably, our analyses suggest elevated transcriptional noise at E3.5 and within the non-committed epiblast at E6.5, coinciding with exit from pluripotency. By contrast, E6.5 primitive streak cells became highly synchronized and exhibit a shortened G1 cell-cycle phase, consistent with accelerated proliferation. Our study systematically charts transcriptional noise and uncovers molecular processes associated with early lineage decisions.

## Highlights

• A high-resolution scRNA-seq map of mouse from peri-implantation to early gastrulation
• Symmetry breaking genes and bivalent chromatin are linked to lineage fate at E4.5
• X chromosome inactivation correlates with *Rlim* and anticorrelates with *Dnmt3a* and *Zfp57*
• Polycomb targets are repressed in the E6.5 epiblast and activated in the primitive streak

## Graphical Abstract

**Figure:**
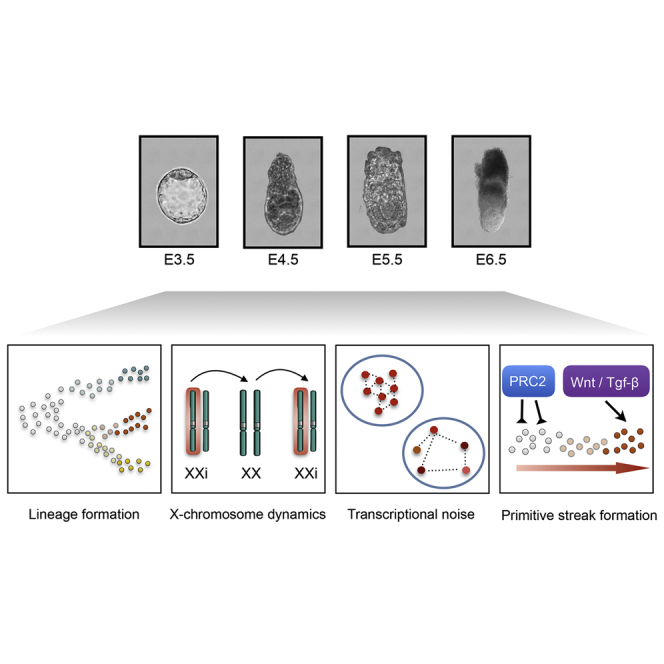

## Teaser

Mohammed et al. chart mouse embryonic development from implantation to early gastrulation at single-cell resolution. They describe regulatory processes associated with lineage commitment. An increased level of transcriptional noise is observed prior to lineage commitment, an observation that provides fresh insights into cell fate decision-making processes.

## Introduction

The peri-implantation mouse embryo consists of the embryonic epiblast combined with two extra-embryonic layers: the trophectoderm and primitive endoderm (PrE). Implantation occurs at approximately embryonic day (E)4.5 and marks several key changes in the embryo (Tam and Loebel, 2007). The pluripotent epiblast dynamically changes post-implantation, developing into a transcriptionally distinct entity primed for differentiation (Boroviak et al., 2015, Nichols and Smith, 2009). This eventually leads to lineage specification at gastrulation, which is initiated by the formation of the primitive streak in a proximal-posterior position (Tam and Loebel, 2007).

Following blastocyst formation, an early milestone in embryonic development is the formation of the epiblast and PrE prior to implantation. This lineage commitment process is largely driven by signaling mechanisms, in particular the Fgf/Mapk signaling pathway (Feldman et al., 1995, Yamanaka et al., 2010). Modulating levels of Fgf directly determines lineage outcome (Kang et al., 2013, Nichols et al., 2009, Yamanaka et al., 2010). Single-cell transcriptome studies recently confirmed a role for Fgf signaling in driving acquisition of PrE identity (Ohnishi et al., 2014). The newly emerging epiblast undergoes a process of transcriptional rewiring and expansion in cell number before further lineage specification is initiated (Boroviak et al., 2015). During this process, the epiblast is subjected to coordinated signaling cues that establish axial polarity (Tam and Loebel, 2007). Wnt, Tgf-b, and Fgf signaling pathways are key drivers of patterning and differentiation, essential for both primitive streak formation and gastrulation (Camus et al., 2006, Conlon et al., 1994, Liu et al., 1999, Mesnard et al., 2006, Sun et al., 1999, Tam and Loebel, 2007, Winnier et al., 1995). Concomitantly, the PrE differentiates further to produce the visceral endoderm (VE), which overlies the epiblast. Regulatory signals from the VE also influence epiblast cell fate, particularly by promoting or antagonizing pathways, such as Wnt and Nodal signaling, in a regionally restricted manner to position and pattern the primitive streak and other embryonic sublineages (Dai et al., 2016, Stower and Srinivas, 2014, Yoon et al., 2015).

A prominent change during these critical stages is the process of X chromosome reactivation and subsequent inactivation in female embryos. The paternally inherited X chromosome, previously inactivated at the 2–4 cell stage, is reactivated in the epiblast during implantation. Subsequently, one of the two X chromosomes is randomly inactivated before the onset of gastrulation (Mak et al., 2004, Okamoto et al., 2004, Schulz and Heard, 2013). The non-coding RNA *Xist*, together with its various regulators, has a pivotal role in silencing the X chromosome (Brockdorff, 2011, Cerase et al., 2015). In vitro, pluripotency-associated transcription factors such as *Pou5f1*, *Nanog*, and *Sox2* are thought to play key roles in reactivation of the X chromosome, in part by downregulating *Xist* transcription (Minkovsky et al., 2012). Recent single-cell studies using embryonic stem cells (ESCs) and epiblast stem cells (EpiSCs) have provided new insights into this process, including the identification of genes potentially involved in X chromosome regulation (Chen et al., 2016). However, a complete single-cell transcriptomic characterization of this process in vivo is lacking. Single-cell analysis in human pre-implantation embryos indicates that X chromosome inactivation is achieved through dosage compensation (Petropoulos et al., 2016).

Single-cell transcriptome studies have been used to examine developmental trajectories and lineage specification in early mouse pre-implantation embryos (Deng et al., 2014, Kurimoto et al., 2006, Ohnishi et al., 2014, Shi et al., 2015) and post-implantation gastrulating embryos (Chen et al., 2016, Scialdone et al., 2016). Several principles underlying cell fate decision-making have been established, including the role of key transcription factor networks, cell signaling, cell position and movement, and mechanical forces (Tam and Loebel, 2007), yet how cells actually transition from one fate to another in vivo is unclear.

Interestingly, uncoordinated transcriptional heterogeneity or transcriptional noise has, on a few specific occasions, been observed to precede cell fate decisions. This heterogeneity has been proposed to aid symmetry breaking (Arias and Hayward, 2006, Eldar and Elowitz, 2010). However, how noise is generated or how precisely it helps symmetry breaking is unknown (Eldar and Elowitz, 2010). Early mouse blastomeres show stochastic transcription of the key transcription factors *Oct4* and *Cdx2*, which become lineage restricted in the inner cell mass (ICM) and trophectoderm, respectively (Dietrich and Hiiragi, 2007). Furthermore, specific PrE and epiblast genes have been shown to be co-expressed in cells within the E3.5 ICM before becoming restricted to specific cell types by E4.5 (Chazaud et al., 2006, Frankenberg et al., 2011, Kang et al., 2013, Morris et al., 2010, Ohnishi et al., 2014, Plusa et al., 2008). A systematic study of transcriptional noise in the developmental stages preceding gastrulation is, however, lacking.

Here, we study the regulatory landscape from peri-implantation to early gastrulation by performing single-cell RNA-sequencing (seq) of mouse embryos from E3.5 to E6.5. Our work provides insights into the exit from pluripotency and priming for differentiation, X chromosome reactivation and subsequent inactivation, and the emergence of regulatory networks associated with cell fate decisions. Our work also investigates transcriptional noise and its sources across the different stages and explores what potential consequences this might have for symmetry breaking and cell fate decision-making.

## Results

### Single-Cell Transcriptomics and Lineage Identification in E3.5, E4.5, E5.5, and E6.5 Embryos

**Figure 1:**
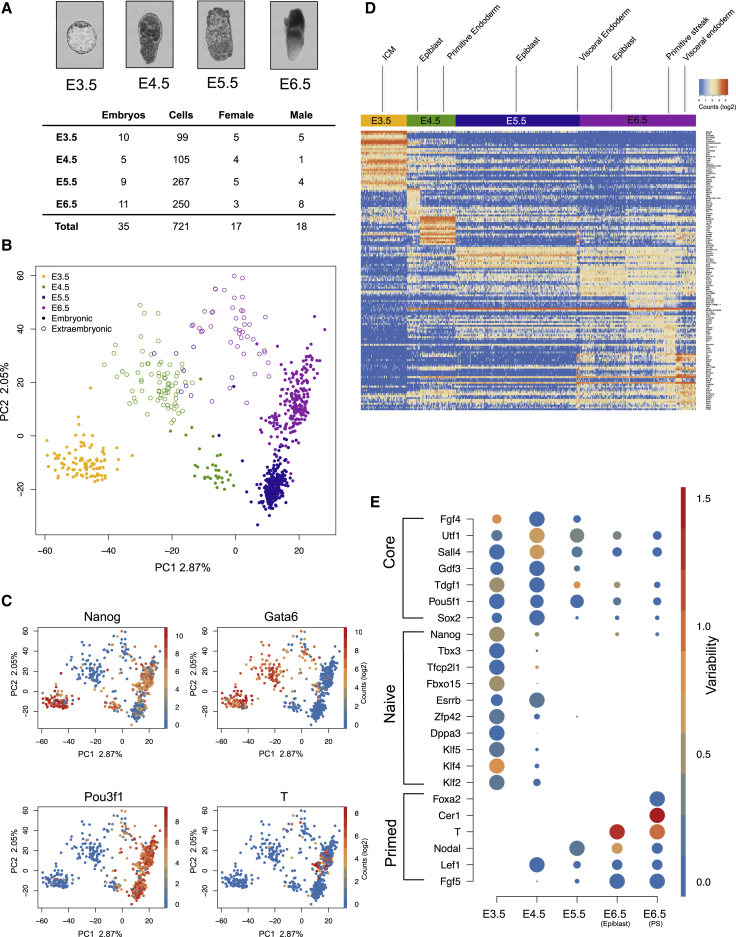
Single-Cell mRNA-Seq of E3.5, E4.5, E5.6, and E6.5 Mouse Embryos (A) Table listing number of embryos and cells, along with representative images of embryos used at each stage. (B) Principal-component analysis (PCA) of cells that passed the quality and filtering criteria. (C) PCA of all cells colored by gene expression levels (log2 of counts per million) of selected marker genes - *Nanog* (ICM/epiblast), *Gata6* (PrE/VE), *Pou3f1* (primed pluripotency), and *T* (primitive streak). (D) Heatmap showing key genes distinguishing cell clusters (SC3 analysis). (E) Gene expression levels and variability of pluripotency factors classified into primed, naïve, and core genes (using previous classifications; Boroviak et al., 2014). The size of each dot represents relative expression levels, while variability is shown by color.

We dissociated embryos into single cells and performed single-cell RNA-seq (scRNA-seq) of mouse pre-implantation ICM at E3.5 and epiblast and extra-embryonic endoderm at E4.5, E5.5, and E6.5. A total of 1,219 cells were manually isolated and sequenced from 32 embryos across all stages, from which 721 remained following filtering for data quality (see Experimental Procedures; Figures 1A and S1A). 128 of the cells were observed during picking to be doublets, but, importantly, including or excluding these samples did not alter our conclusions (Figure S1B). Transcriptomes derived from isolated cells were subjected to principal-component analysis (PCA) showing that the dataset separates by developmental stages and lineage, consistent with known marker-based lineage identities (Tam and Loebel, 2007) (Figures 1B and 1C).

To rigorously interrogate lineage identities and associated gene markers, we employed single-cell consensus clustering (SC3) (Kiselev et al., 2017) using all expressed genes, as well as subsets of non-coding RNAs and transcription factors (Figures 1D and S1C). This identified eight clusters of cells and associated marker gene sets, which distinguished embryonic and extra-embryonic cells and additionally identified four subclusters within the E6.5 embryo. Consistent with the PCA, E3.5 cells do not possess distinct lineage identities, as previously reported (Ohnishi et al., 2014). Networks of genes including several known naive pluripotency markers are observed exclusively at this stage. At E4.5, a clear separation of cells into the epiblast and PrE is observed and characterized by exclusive expression of known markers, such as *Nanog*, *Esrrb*, *Fgf4* (epiblast) and *Gata6*, *Pdgfra*, and *Sox17* (PrE) (Figure S1D). The E5.5 epiblast cells cluster separately from E4.5 epiblast cells and possess reduced *Nanog* expression, while gaining primed pluripotency markers such as *Pou3f1*. Cells within the E5.5 epiblast do not show any apparent substructure. Finally, cells from the E6.5 embryo cluster into four groups corresponding to the VE, a primitive streak population and two subclusters of non-committed epiblast cells (epiblast 1 and epiblast 2). The primitive streak is identified by high *T* expression in addition to the presence of *Snai1*, *Lef1*, *Evx1*, and *Mesp1*. We also identify several additional markers for the streak including *Greb1*, *Ncoa5*, *Eid1*, and *H2afy2*. A list of lineage-specific genes can be found in Table S1, along with differential expression of lineages in Table S2. Gene pathway analyses indicate an increasing role for signaling pathways such as Wnt, Bmp, Fgf, and Nodal during development, with the primitive streak population exhibiting high pathway activity (see Experimental Procedures; Figure S1E).

In terms of charting pluripotency across the stages, E3.5 exhibits the highest levels of naive pluripotency followed by E4.5. At E5.5 and E6.5, embryos no longer express naive factors and switch to primed pluripotency markers (Figure 1E) (Boroviak et al., 2015). E4.5, E5.5, and E6.5 epiblast cells show remarkable differences in their expression of pluripotency components, while maintaining a relative lack of evident differentiation, which potentially suggests a shift toward a “formative” state of pluripotency prior to differentiation (Kalkan and Smith, 2014). Interestingly, we found that many of these markers are not homogenously expressed in the embryos. For example, at E3.5, *Klf4* and *Fgf4* are variably expressed as are *Nanog*, *Sall4*, and *Utf1* at E4.5 and *T* and *Cer1* at E6.5 (Figure 1E).

### Reactivation and Subsequent Inactivation of the X Chromosome

**Figure 2:**
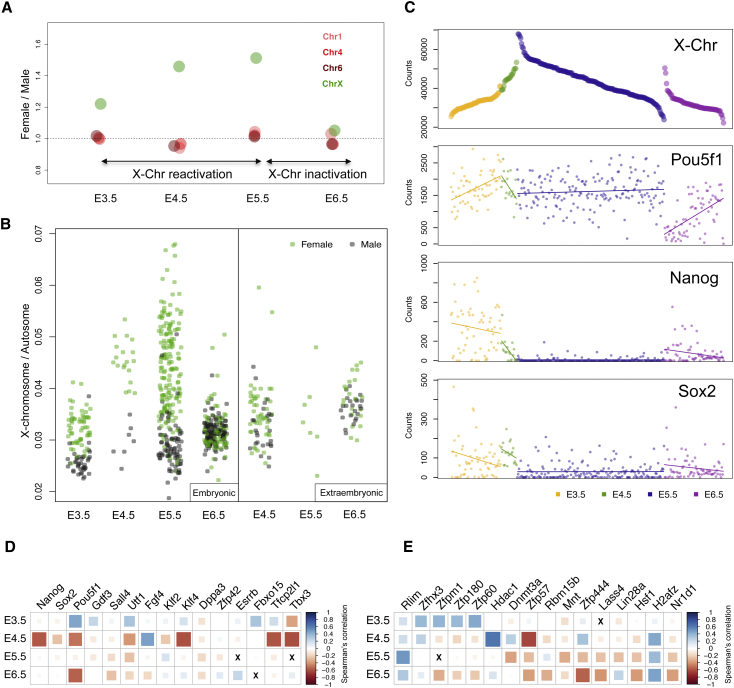
Dynamics of X Chromosome Reactivation and Silencing (A) Ratio of gene expression (median) between female and male embryos for the X chromosome and chromosomes 1, 4, and 6. (B) Total expression levels of the X chromosome visualized at a single-cell level. The y axis represents the proportion of X chromosome counts relative to all other chromosomes. (C) Overall X chromosome expression levels plotted across all stages. The matched expression levels for *Pou5f1*, *Nanog*, and *Sox2* are also plotted underneath. (D) Plot representing pluripotency genes correlating or anticorrelating with X chromosome expression at each stage. Spearman’s correlation coefficient is represented by color and size. (E) Plot representing selected genes correlating or anticorrelating with X chromosome expression at each stage. The X represents absence of expression.

The presence of multiple embryos of both sexes enabled us to investigate potential gender-based differences in early development. In particular, the process of reactivation and subsequent inactivation of the female X chromosome was investigated in detail. Gender was assigned to each embryo by measuring the expression of genes on the Y chromosome (see Experimental Procedures; Figure S2). Comparison of gene expression ratios between males and females from the X chromosome and autosomes reveals increased X chromosome expression in females starting at E3.5 (Figure 2A). This difference is more noticeable at E4.5 and E5.5, where both X chromosomes become fully activated. Inactivation of the X chromosome is initiated at E5.5, and by E6.5, X chromosome expression levels in female embryos were reduced to levels comparable to those of other chromosomes. Analysis of X chromosome expression relative to autosomes at the single-cell level reveals the heterogeneous nature of this process within a given stage (Figure 2B). Additionally, transcriptome data at the single-cell level allows separation of embryonic and extra-embryonic cells: extra-embryonic endoderm cells do not reactivate the paternal X chromosome (Kobayashi et al., 2016), and, thus, both male and female cells express similar levels of X chromosome genes.

Consequently, we explored the correlation between gene expression levels and X chromosome activation status in the embryonic lineage to identify genes associated with this process and to characterize the relationship with known regulators. The pluripotency factors Pou5f1, Nanog, and Sox2 are thought to be key regulators of X reactivation (Donohoe et al., 2009, Navarro et al., 2008). Consistent with this, we found (Figure 2C) that X chromosome reactivation is positively correlated with *Pou5f1* expression levels at E3.5 (Spearman correlation = 0.45), albeit, the result is not significant after correcting for multiple testing (Table S3). Counter-intuitively, *Pou5f1* was negatively correlated with X chromosome levels after inactivation (Spearman correlation = −0.54, adjusted p = 9.75 × 10^−05^). However, since cells in the primitive streak express higher *Pou5f1* levels, this association may reflect lineage-based expression differences rather than X chromosome inactivation. In contrast, *Nanog* and *Sox2* show no clear correlation with either the reactivation or inactivation process. In addition to evaluating the roles of known pluripotency regulators, several other candidates were identified (Figures 2D and 2E). The top 100 genes identified as being associated (positively or negatively correlated) with reactivation include the *Pou5f1* interacting transcription factor *Zfhx3* and several zinc-finger proteins such as *Zfpm1*, *Zfp180*, and *Zfp60* (Table S3).

X inactivation analysis was performed at E5.5, as the process of inactivation begins at this stage and the analysis can therefore reveal potential drivers of inactivation. Among the top genes associated with X chromosome inactivation at E5.5 were the DNA methyltransferase *Dnmt3a* (Spearman correlation = −0.35, adjusted p = 0.0006) and the transcriptional repressor *Zfp57* (Spearman correlation = −0.27, adjusted p = 0.02). *Dnmt3a* and *Zfp57* have been shown to interact with each other, and *Dnmt3a* has been shown to regulate *Xist* expression (Quenneville et al., 2011). It is particularly noteworthy that *Zfp57* expression is negatively correlated with X chromosome levels at both X reactivation (Spearman correlation = −0.62) and X inactivation. Inactivation is furthermore correlated with several other genes including chromatin and transcription regulators such as *Mnt*, *Zfp444*, *Lass4*, *Lin28a*, *Hsf1, H2afz*, and *Nr1d1* (Table S3). The heat-shock transcription factor *Hsf1* has been previously implicated in inactivating X and Y chromosomes during meiotic repression in spermatogenesis (Akerfelt et al., 2010), as has the histone *H2afz* substitution on the X chromosome (Sin et al., 2012), suggesting a role for these proteins in X chromosome inactivation. X-inactivation at E5.5 was also correlated with a decrease in expression of *Rlim*, a ubiquitin ligase that has been previously implicated in regulating X chromosome inactivation (Shin et al., 2010).

### Systematic Assessment of Transcriptional Noise across Early Development

Transcriptional heterogeneity within a tissue may indicate the presence of coordinated transcriptional networks associated with the presence of different cell types. By contrast, transcriptional variability occurring in the absence of cell-type substructure indicates the existence of uncoordinated transcriptional heterogeneity or transcriptional noise. We explored approaches to quantifying global levels of transcriptional noise across the different stages of embryo development, focusing on the embryonic lineage. We selected homogeneous cell populations as a first step. Epiblast cells from E3.5 to E5.5 do not show any substructure, however, at E6.5, cells start to differentiate into the primitive streak. To ensure that transcriptional heterogeneity is not driven by the presence of cell subpopulations, we separated E6.5 primitive streak cells and E6.5 epiblast cells. We specifically selected cells from the uncommitted anterior epiblast by estimating their most likely original position in the embryo using the transcriptomic profile of the four quadrants of the E6.5 epiblast (anterior-proximal, anterior-distal, posterior-distal, and posterior-proximal) (Wu et al., 2015).

**Figure 3:**
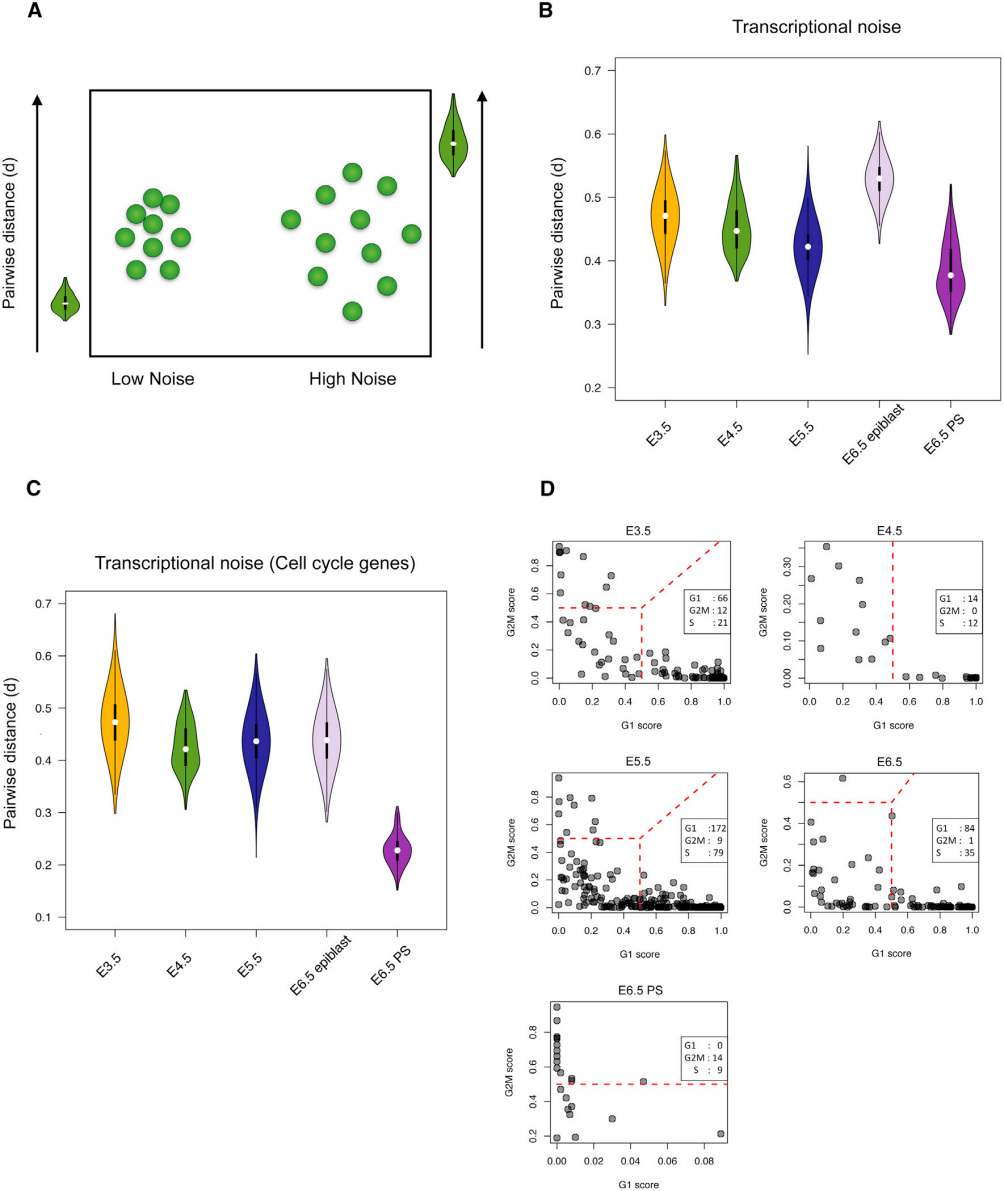
Transcriptional Noise across Each Developmental Stage (A) Cartoon illustrating the concept used to calculate transcriptional noise. The method is based on cell-cell correlations within homogeneous populations. (B) Plot showing transcriptional noise across all stages of epiblast development and the primitive streak (PS). (C) Plot showing transcriptional noise of cell-cycle genes across all stages of epiblast development and the primitive streak (PS). (D) Separation of cells at each stage or lineage by cell-cycle phase. Number of cells at each cell-cycle stage is shown in the inset.

The measure of transcriptional noise used here is based on pairwise cell-cell distances (d) (see Experimental Procedures). As noise levels increase, cells become more dispersed in the high-dimensional gene expression space, thus increasing the average pairwise distance. By contrast, when noise levels decrease, cells tend to be more similar to each other and d decreases (Figure 3A). The results suggest that transcriptional noise is higher (p < 0.0001) in the uncommitted ICM at E3.5 than in the committed epiblast at E4.5 (Figure 3B). We also observed a decrease in noise within the primitive streak, further suggesting that lineage differentiation may be accompanied by a decline in transcriptional noise. Interestingly, we observed an increase of transcriptional noise in the uncommitted epiblast at E6.5 in comparison to the E5.5 epiblast, coinciding with the exit from pluripotency. Importantly, our findings were consistent across the different batches that correspond to different litters, giving confidence in the method and biological origin of this finding (Figures S3A and S3B). We validated our findings at E6.5 using an independent dataset (Scialdone et al., 2016) from a different mouse strain. The results confirmed that transcriptional noise in the uncommitted epiblast was significantly higher (Wilcoxon test, p < 0.0001) than in the primitive streak (Figure S3C).

We next explored if heterogeneity with respect to cell-cycle stages was linked with the observed levels of transcriptional noise. We computed cell-cell distances (d) considering only genes associated with cell-cycle regulation (KEGG 0411004110). We found a slight increase in cell-cycle heterogeneity at E3.5 versus E4.5 (Figure 3C), however, the heterogeneity of cell-cycle genes in E5.5 and E6.5 epiblast were comparable, indicating that the increased global noise in the epiblast cells at E6.5 is not driven by cell-cycle genes. There was a striking drop of cell-cycle heterogeneity in the primitive streak, suggesting that these cells are far more synchronized than at any other stage. To further investigate this, we assigned a cell-cycle stage to each cell (Scialdone et al., 2015), which revealed a mixture of cell-cycle stages in embryos from E3.5 to E5.5 with a high proportion of cells in the G1 phase (Figure 3D). By contrast, the E6.5 primitive streak cells were substantially enriched for the G2/M stage with few cells in the G1 phase, providing a molecular correlate of their significantly accelerated cell division rate (O’Farrell et al., 2004, Snow, 1977).

### Symmetry Breaking in the Epiblast and PrE Transition

**Figure 4:**
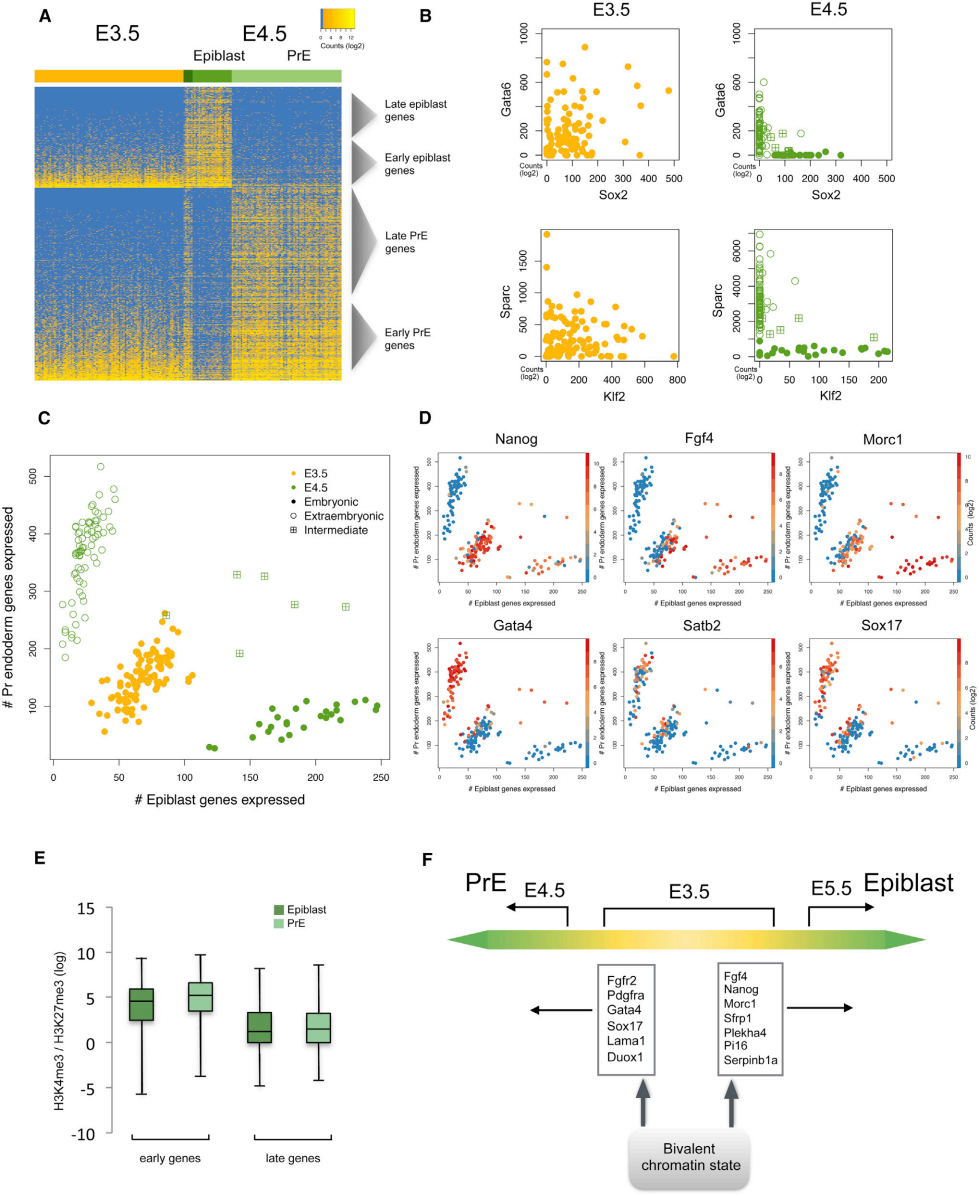
Transcriptional Noise during Primitive Endoderm and Epiblast Formation (A) Heatmap showing expression of PrE and epiblast genes identified by differential expression at E4.5. A substantial number of genes are already expressed at E3.5, but genes do not segregate by lineage at E3.5. (B) Examples of PrE and epiblast genes at E3.5 and E4.5 indicating an initial co-expression before becoming lineage specific at E4.5. (C) Plot representing number of lineage-specific genes expressed in each E3.5 and E4.5 cell. (D) Overlay of expression levels of genes identified as correlated with the ratio of epiblast and PrE expressed genes. (E) Plot representing the ratio of H3K4me3 and H3K27me3 enrichment at binding sites classified as early or late lineage genes. Early genes show a higher ratio than their late counterparts (p < 0.001). (F) Cartoon showing the overall transition from a non-committed state to lineage commitment and genes associated with this shift.

It is known that prior to the emergence of coherent transcriptional networks that define epiblast or PrE identity at E4.5, specific endoderm or epiblast genes at E3.5 are first co-expressed, then separate into a “salt and pepper” pattern before forming the PrE and epiblast (Chazaud et al., 2006, Ohnishi et al., 2014, Plusa et al., 2008). This pattern allows the co-expression of opposing lineage markers in an uncoordinated fashion before lineage exclusive expression. To explore this transition in detail, we investigated lineage segregation from E3.5 to E4.5. At E4.5, the cells segregate into two clear subpopulations, the epiblast and the PrE. Differential expression analysis between these two cell subgroups at E4.5 identified 689 genes enriched for the PrE and 382 genes in the epiblast lineage (adjusted p value ≤ 0.05, log2 fold change > 2) (Table S2). Although lineage subgroups are not observed at E3.5, specific subsets of PrE and epiblast genes are already expressed. This suggests the existence of an “early” (E3.5) and “late” (E4.5) wave of lineage expression as previously described (Plusa et al., 2008) (Table S4). Consistent with these observations, both epiblast and PrE markers, such as *Nanog/Sox2* and *Gata6/Sox17*, were expressed in a non-lineage-based random manner at E3.5 (Figure 4A), exhibiting substantial co-expression before displaying mutually exclusive lineage-specific expression patterns at E4.5 (Figure 4B). At E3.5, the non-coordinated expression of epiblast and PrE markers contributes to the lack of distinct lineage identity. Interestingly, a small subpopulation of cells at E4.5 also co-expresses both lineage markers (Figures 4A, S4A, and S4B). These “intermediate” cells, however, unlike E3.5 cells, express both early and late markers and were excluded from subsequent analyses. Prior visual annotation of the cells excludes the possibility that these samples were doublets.

Using the above defined lineage genes, E3.5 and E4.5 cells were analyzed to determine the ratio of PrE or epiblast genes expressed in each cell (Figure 4C). E3.5 cells expressed both lineage markers, while, as expected, the cells at E4.5 were strongly biased toward one lineage or the other. To address the question of how cells break the “noisy symmetry” at E3.5, we identified genes with expression levels that are correlated with the ratio of epiblast to PrE genes, hypothesizing that such genes might represent key drivers of lineage commitment (Figures 4D and S4C). Using this method, we identified *Fgf4* and *Fgfr2*, which were previously shown to be important in driving PrE and epiblast specification, respectively (Ohnishi et al., 2014), providing confidence in our approach. Notably, we found *Pdgfra*, *Top2b*, *Sox17*, *Gata4*, and *Pdk2* as being associated with a shift toward the PrE cell fate. Similarly, *Morc1*, *Nanog*, *GM10664*, *Dppa5a*, and *Pdpn* were associated with the epiblast fate, providing a rich source of unique candidate lineage driver genes. These drivers are candidates for genes that are upstream of known lineage identity genes, and this concept can be tested in future functional experiments. Analysis of individual E3.5 embryos indicated that this break in symmetry does not reflect developmental differences between individual embryos (Figure S4D).

We next explored potential underlying regulatory mechanisms driving lineage specification using EnrichR (Chen et al., 2013) gene set enrichment analysis. The analysis was used to compare the PrE and epiblast gene sets against existing chromatin immunoprecipitation (ChIP)-seq datasets to identify potential master regulators. The results indicated that epiblast genes were potentially regulated by pluripotency factors, such as Nanog and Pou5f1 (Table S4). Both epiblast and PrE genes showed a significant enrichment for binding by polycomb components, such as Ezh2 and Suz12. Developmental genes are thought to be regulated by bivalent chromatin marks (Voigt et al., 2013) (H3K27me3 and H3K4me3), and, hence, we analyzed occupancy by bivalent chromatin marks at both PrE and epiblast genes using regions defined in ESCs (Rugg-Gunn et al., 2010). Both PrE and epiblast genes showed enrichment for bivalency (Figure S4E), which increased in the late lineage defining genes. To investigate underlying differences between bivalent chromatin marking the early noisily expressed lineage genes and the more coherent late lineage genes, we quantified H3K4me3 and H3K27me3 marks at these early and late lineage genes using ChIP-seq datasets from the ICM (Zhang et al., 2016, Zheng et al., 2016). We analyzed the ratio of H3K4me3 to H3K27me3 (Figure 4E) at each site and found significantly (p < 0.0001) increased H3K4me3 levels at the early lineage genes when compared to the late genes, which were relatively more enriched for H3K27me3. This shows that late lineage genes in the ICM (where they are repressed) are bivalent, but with higher H3K27me3 levels than early genes (which are active) (Figure 4F). Bivalency in active genes (as in the E3.5 ICM) has recently been linked with increased transcriptional noise (Kar et al., 2017).

### Exit from Pluripotency and Lineage Commitment

The transition from E5.5 to E6.5 is characterized by increased transcriptional noise in the uncommitted epiblast portion, along with a striking decrease in noise within the primitive streak (Figure 3B). A decrease in expression of naive pluripotency-associated genes is also observed along with gain in primed pluripotency-associated genes (Figure 1E). Despite expression of signaling components associated with anterior-posterior polarity and the primitive streak, such as *Cripto*, *Nodal*, and *Lefty1*, the epiblast at E5.5 shows no apparent substructure or expression of primitive streak markers, suggesting that only a handful of key regulatory molecules might be asymmetrically expressed and sufficient for patterning asymmetries at this stage.

**Figure 5:**
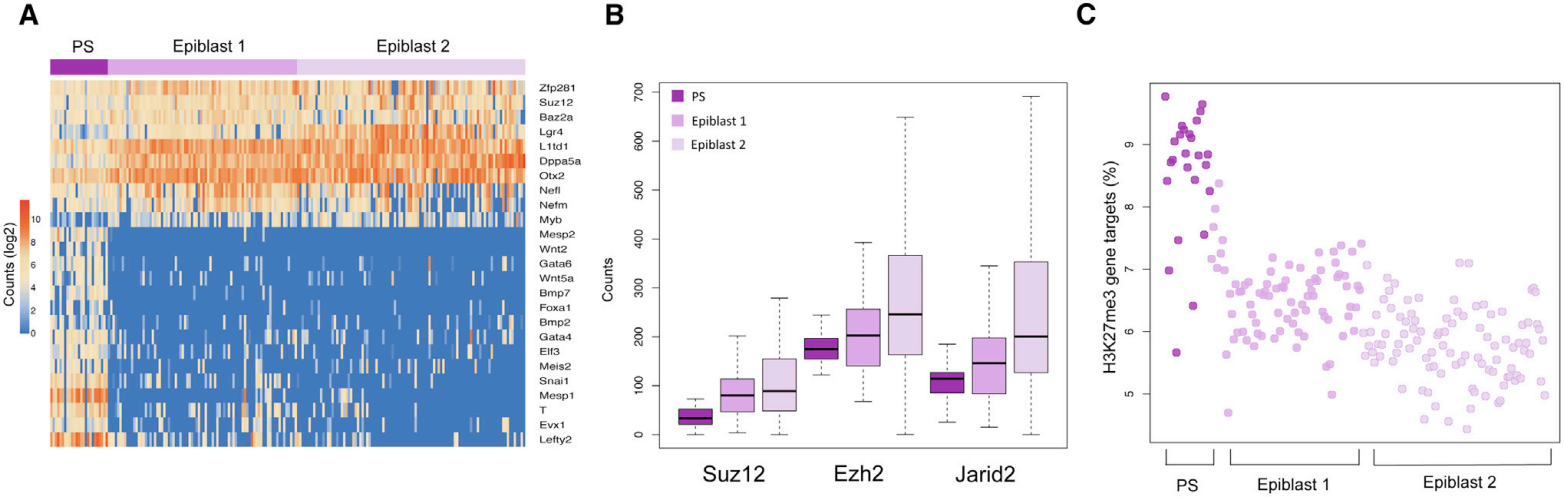
Lineage Characteristics at E6.5 (A) Heatmap showing the expression of some differentially expressed genes between the primitive streak cluster and the epiblast. (B) Expression levels of polycomb components *Suz12*, *Ezh2*, and *Jarid2* across the different epiblast subclusters and primitive streak (counts per million). (C) Plot representing H3K27me3 bound genes expressed in E6.5 epiblast and primitive streak cells using published in vivo datasets (Zylicz et al., 2015).

By SC3 clustering of E6.5 cells, an initial embryonic and extra-embryonic distinction was observed. Extra-embryonic VE cells could be further stratified to identify the anterior VE (AVE) using the known markers *Hhex*, *Cer1*, *Lefty1*, and *Dkk1* (Figure S5A). By examining genes with expression patterns correlated with *Cer1*, additional markers such as *CD24a* and the Wnt antagonist *Sfrp1* were associated with the AVE, while several genes, such as *Tdh*, *Sord*, and the Wnt-associated gene *Srebf2* were enriched in the non-AVE population. We also performed differential expression analysis between the two groups of cells to identify additional markers (Table S5). The epiblast consists of three main subgroups, a primitive streak population and two epiblast subclusters (Figure 5A). These two epiblast subclusters show only small differences in the expression of genes, such as the Wnt pathway receptor *Lgr4* and thus may represent cells only slightly separated in developmental time or space. *T* positive cells in the E6.5 embryo are observed to be both *Mesp1* positive and *Mesp1* negative, as previously described (Figure S5B) (Scialdone et al., 2016). Genes upregulated in the primitive streak subcluster include known primitive streak and mesendodermal markers, such as *Mesp1*, *Mesp2*, *Bmp2*, *Snai1*, *Gata4*, and *Gata6* (Table S5) and also several additional markers, such as *Greb1*, *H2afy2*, *Eid1*, and *Ncoa5*. Interestingly, we identified consensus motifs for Mixl1, Pou5f1, and Foxh1 in the promoter regions of primitive streak genes (Figure S5C), suggesting that these transcription factors may drive the establishment of mesendodermal fate. Within the epiblast, there was a notably increased expression of the exit from pluripotency-factor *Otx2*, along with several polycomb genes, including *Suz12*, *Ezh2*, and *Jarid2* (Figure 5B). Indeed, analysis of H3K27me3 ChIP-seq data from the E6.5 epiblast (Zylicz et al., 2015) shows that polycomb bound genes were significantly upregulated in the primitive streak compared to the rest of the epiblast (Figure 5C). This suggests that the polycomb complex has an important role in establishing transcriptional control in the non-committed epiblast cells, including regulating the transition to form the primitive streak, consistent with the phenotype observed in Eed knockout embryos (Faust et al., 1998).

## Discussion

In this study, we applied single-cell RNA-seq at a detailed level of resolution to explore early mouse development from pre-implantation to early gastrulation. Besides this valuable resource for the community, our study makes a number of important observations. First, we have identified several candidates for lineage driver genes, both at E4.5 and at E6.5. Second, we charted major epigenetic and signaling events across the exit from pluripotency leading up to gastrulation. Third, we mapped X chromosome reactivation and subsequent inactivation dynamics together with identifying candidate regulators for both steps. Fourth, we systematically explored the concept of transcriptional noise across developmental stages and showed this is highest in uncommitted cells and subsides upon commitment and differentiation. Fifth, we mapped cell-cycle stage composition at each developmental time point and discovered a striking synchronization of the cell cycle in the primitive streak.

Single-cell sequencing technologies have allowed detailed investigation of complex heterogeneous systems, including early development (Kumar et al., 2017). However, with a capture efficiency ranging between 10% to 50% (Islam et al., 2014), the low amount of starting material renders these approaches susceptible to technical artifacts and batch effects (Tung et al., 2017). Additionally, highly expressed genes are more reproducibly recovered and hence methodologies that address and account for these problems, such as correcting for technical batches and establishing stringent quality parameters need to be implemented, as we have done in this study.

Overall the kinetics of X reactivation (E3.5 and E4.5) and inactivation (E5.5 and E6.5) we observed are consistent with fluorescent reporter-based studies (Kobayashi et al., 2016), lending added confidence to our chromosome-wide observations. Single-cell resolution allows the dynamics of this process to be studied in individual embryos, revealing heterogeneity within embryos and thus identifying genes linked with X reactivation or inactivation.

A recent study using in vitro models identified several candidate regulators of the X-inactivation process and also concludes that pluripotency and X-inactivation are not tightly linked (Chen et al., 2016). In vitro studies cannot capture the full extent of heterogeneity within embryos nor recapitulate embryonic stages and transitions. Pluripotency factors, particularly *Pou5f1*, *Sox2*, and *Nanog*, are known to downregulate *Xist* in ESCs and hence are prime candidates for X reactivation in vivo (Navarro et al., 2008, Navarro and Avner, 2009). A more recent study has questioned how direct this link is (Minkovsky et al., 2013), suggesting the potential involvement of other regulatory factors, which our single-cell association analysis is able to uncover. We found a strong association between X reactivation and *Pou5f1* expression (but not with *Sox2* and *Nanog*) at E3.5, which quickly weakened, suggesting that other factors may also mediate or maintain the reactivated state, including the *Pou5f1* interacting partner *Zfhx3*. The following period of rapid X-inactivation was associated with increased expression of *Dnmt3a* and *Zfp57*, reinforcing the importance of DNA methylation in random X-inactivation (Auclair et al., 2014). Interestingly, *Zfp57* expression levels are also associated with the reactivation process, being anticorrelated with X chromosome expression levels. This suggests that *Zfp57* may act as repressor of the X chromosome during these stages of development. Consequently, the sets of genes identified in our study provide additional insights into the mechanisms underpinning X-inactivation and reactivation and are strong candidates for future functional studies.

An intriguing observation in our study is the different levels of transcriptional noise found at each stage. Significant differences in noise were detected in cell populations without a coherent substructure; i.e., without different lineages or cell types being present. Previous studies analyzing specific genes have suggested stochastic transcription of *Oct4* and *Cdx2* preceding their lineage-resolved expression in ICM and trophectoderm (TE), respectively (Dietrich and Hiiragi, 2007), and the same is true of some PrE and epiblast genes in the E3.5 ICM before becoming restricted to the specific cell types by E4.5 (Ohnishi et al., 2014). Here, we show that this is globally true of the E3.5 to E4.5 transition. Furthermore, the primitive streak shows much reduced noise in comparison to the uncommitted epiblast at E6.5. These results suggest the possibility of a general principle for increase of transcriptional noise prior to lineage differentiation.

Noise in gene expression has been suggested to feed directly into protein expression (Bar-Even et al., 2006, Newman et al., 2006) and importantly is linked with lineage commitment in other systems. In hematopoietic stem cells, gene expression noise has been shown to create heterogeneity in expression of lineage drivers, which in turn directly influences the availability of certain lineage fates (Chang et al., 2008). Using a single-cell approach, candidate erythroid progenitors have been linked with increased transcriptional noise prior to differentiation (Richard et al., 2016). Transcriptional noise has also been attributed to regulating the reactivation of latent HIV (Dar et al., 2014). In terms of possible mechanisms, mixed-lineage states have recently been reported in hematopoietic precursors where opposing transcription factors were suggested to compete with one another for binding to the DNA prior to establishing more defined lineages (Olsson et al., 2016). Indeed, in early embryos, Nanog and Gata6 have such opposing roles (Chazaud et al., 2006, Frankenberg et al., 2011, Fujikura et al., 2002, Shimosato et al., 2007), with in vitro assays suggesting direct repression of the *Gata6* promoter by the Nanog protein (Singh et al., 2007). Therefore, higher levels of noise observed at E3.5 might arise from such mutual antagonism, thereby blocking specification of either lineage. The polycomb system through bivalent domains (Wassef et al., 2015) and the DNA methylation/demethylation system (Lee et al., 2014) are also potential sources of transcriptional noise.

Interestingly, the genes identified as pioneering symmetry breaking from E3.5 are primarily transcription factors such as *Sox17*, *Nanog*, *Gata4*, and *Satb2* (Figure 4D). Overexpression of *Gata4* or *Sox17* (McDonald et al., 2014, Shimosato et al., 2007) in ESCs is sufficient to direct cells toward a PrE-like state, suggesting that tipping the transcriptional balance is important for lineage decisions. We show that the PrE and epiblast genes are enriched for bivalent chromatin domains in the E3.5 ICM, with late (i.e., lineage specific) genes having relatively higher levels of H3K27me3, suggesting that a changing ratio of H3K4me3 to H3K27me3 may in part be responsible for the transition from noisy to lineage-resolved expression.

The establishment of anterior-posterior (AP) polarity precedes the formation of the primitive streak and is first evident by the spatially restricted activity of signaling pathways, such as Nodal, Wnt, and Fgf. Strikingly, at E6.5, as lineages start to be specified, clear signaling pathway enrichment for BMP, Wnt, Nodal, and Fgf is evident in our data (Figure S1E). Hence, the elevation of noise in the uncommitted epiblast at the E6.5 stage may create “competence” for subpopulations of cells to respond to signaling systems (e.g., Nodal and Wnt inhibitors in the AVE), thereby helping to break symmetry.

Finally, our dataset allowed identification of cell-cycle phases for each lineage and stage, with substantial differences observed. Cells in the primitive streak are thought to cycle very rapidly, which may be achieved by a shortened or non-existent G1 phase (O’Farrell et al., 2004). Indeed, the E6.5 primitive streak demonstrates a distinct cell-cycle signature with a large proportion of cells in the G2/M and S phase. Signaling factors, in particular Wnt signaling, are thought to interface with cell-cycle regulation (Davidson and Niehrs, 2010). This potentially initiates synchrony between cells, which in turn may direct the low levels of noise observed. The resulting coherence may thus be important in maintaining the precision of lineage commitment programs in the gastrulating embryo.

Our work reveals large-scale modulation of transcriptional noise across the different stages of early development. High noise may establish a special state of developmental competence so that cells can respond to internal or external cues in a more flexible and refined way in order to break symmetry. The underlying causes and regulators of noise potentially differ according to stage and context. Future studies should be aimed at identifying such regulators together with their manipulation in vivo, to better understand the biological significance of this intriguing phenomenon.

## Experimental Procedures

### Preparation of Single Cells from Early Embryos

All experimental procedures were performed in accordance with the Animals (Scientific Procedures) Act 1986 and by local authority granted by the Animal Welfare and Ethical Review Body (AWERB) committee of the Babraham Institute. Blastocysts were isolated from C57BL/6Babr mice following inter se mating, at E3.5 or 4.5. E3.5 blastocysts were flushed from the uterus using M2 medium and the zonae removed by short incubation in acid tyrodes solution. Embryos at E4.5 were dissected from nascent decidua. The trophectoderm was eliminated from E3.5 and E4.5 embryos by immunosurgery (Solter and Knowles, 1975). Embryos were incubated for 15–30 min in 20% anti- mouse antiserum in N2B27 (made in house), rinsed three times in N2B27, and transferred to 20% freshly thawed rat serum in N2B27 for 15–30 min. Lysed trophectoderm was removed manually by repeated aspiration using a mouth-controlled, finely drawn, Pasteur pipette just bigger than the ICM. Isolated ICMs were placed individually in 20 μL drops of Accutase in a bacteriological dish for at least 5 min until they assumed a blackberry-like appearance. They were transferred to similar sized drops of M2 and aspirated repeatedly using a very finely drawn Pasteur pipette to separate the cells. Cells were immediately placed individually into wells of lysis buffer and frozen. E5.5 embryos were dissected manually and were observed to be early E5.5 cells lacking distinct thickening of the VE (pre-AVE migration). The extra-embryonic ectoderm was removed using the tip of a pulled Pasteur pipette. The epiblast was separated from overlying VE by aspiration, distal end first, with a pulled Pasteur pipette with a diameter just bigger than the epiblast. Both epiblast and VE were separately disaggregated and plated into wells as described above for ICMs.

E6.5 embryos were visually staged as early streak (Downs and Davies, 1993) and dissected from decidua in PBS and placed into M2 solution to remove extra-embryonic tissues. Differences in dissection may contribute to different proportions of primitive streak and epiblast cells obtained at E6.5. The embryonic ectodermal portion of the early streak embryo was dissociated in drops of TrypLE at room temperature and upon partial digestion was fully dissociated by repeated aspiration using a very finely drawn, mouth-controlled, siliconised-glass needle. Cells were assigned as single, doublets, or multiples by visual inspection and placed directly into lysis solution and frozen prior to library generation.

### RNA-Processing and Library Preparation, Sequencing

mRNA from isolated single cells was isolated and amplified using the bead-based capture and amplification previously described in the G&T-seq protocol (Macaulay et al., 2015). Multiplexed sequencing libraries were generated from cDNA using the Illumina Nextera XT protocol and 100 bp paired-end sequencing was performed on an Illumina HiSeq 2500 instrument.

Sequences were trimmed using trim_galore (v0.4.1, http://www.bioinformatics.babraham.ac.uk/projects/trim_galore/) using default settings. Trimmed data were separately mapped to the mouse GRCm38 genome assemblies using HISAT2 (v.2.0.1_Beta) with options-sp 1000,1000-no-mixed-no-discordant, and was filtered to remove non-primary alignments or alignments with mapping quality (MAPQ) < 20. Mapped RNA-seq data were quantitated using the RNA-seq quantitation pipeline in SeqMonk software (http://www.bioinformatics.babraham.ac.uk/projects/seqmonk/). Genome version used was *Mus musculus* GRCm38 (08/06/2012).

### Data Filtering

Cells annotated as triplets, multiples, or empty wells were filtered out. Cells expressing fewer than 4,000 genes or more than 10% of mapped reads allocated to mitochondrial genes were removed in quality control. Furthermore, 49 trophoblast cells were identified by the expression of Elf5, Wnt7b, and Tex19.1 markers (more than one count in each gene) and were not considered in downstream analysis. Out of the 1,219 cells that were captured across the experiment, 721 passed our quality and filtering criteria.

### Data Analysis

Gene expression levels were normalized in terms of reads per million of mapped reads to the transcriptome of each cell. We only considered genes on the autosomal chromosomes except for the X chromosome analysis, where all genes were used for the normalization. PCA analysis was performed on the normalized counts, excluding genes expressed in less than three cells. Gene pathway annotations were obtained from ConsensusPathDB (http://www.cpdb.molgen.mpg.de) (Table S1). Variability of each gene was calculated by fitting the squared coefficient of variation as a function of the mean normalized counts and then calculating the distance to rolling median. Correlation analyses were performed excluding non-expressed genes and using Spearman’s correlation as a measure of relationship between variables.

DESeq2 R library (Love et al., 2014) was used for differential expression analysis. Genes were identified by having a log2 fold change significantly greater than 1 at a false discovery rate threshold equal to 0.05. Due to the high number of genes differentially expressed between epiblast and PrE at E4.5, we used a log2 fold change threshold of 2.

For sex determination, embryos with a chromosome-Y count sum fewer than ten were classified as female and the rest of the embryos were classified as males. The expression levels of genes on the X chromosome were summed and used to perform Spearman correlation analysis. For the correlation comparisons, we used all genes with mean expression >1 in each of the stages. P values were corrected for multiple testing by using the false discovery rate (FDR) method.

ChIP-seq datasets for H3K4me3, H3K27me3 in ESCs, and H3K27me3 in E6.5 embryos were obtained from existing studies Rugg-Gunn et al. (2010) and Zylicz et al. (2015), respectively. Datasets for H3K4me3 and H3K27me3 (Zhang et al., 2016, Zheng et al., 2016) were used to quantify bivalency. All H3K4me3 bound regions were first selected and levels of H3K27me3 were quantified across the different gene sets. Statistical significance was determined using a Wilcoxon signed rank test. We used DREME in conjunction with TOMTOM (Bailey et al., 2009) to identify discriminatory transcription factor binding sites between the promoters (500 bp upstream of the transcription start site [TSS]) of the primitive streak and epiblast cell clusters.

### Transcriptional Noise

Technical batch effects within a lineage of a given stage were first corrected using ComBat (Leek, 2014). We selected cells from the epiblast lineage at E4.5 and E5.5 from our clustering method. For cells at E6.5, we estimated their most likely original position in the embryo by using a dataset of the transcriptomic profile of the four quadrants of the E6.5 epiblast (Wu et al., 2015). This information was used in addition to our clustering analysis in order to distinguish an uncommitted set of E6.5 epiblast cells (those mapping to the anterior-distal region) from a group of E6.5 cells already committed to a mesendodermal fate, as determined by their gene expression pattern and their posterior-proximal inferred position. The number of cells used for each stage were: E3.5: 99, E4.5: 26, E5.5: 260, E6.5 epiblast: 120, and E6.5 primitive streak: 23.

We then identified highly variable genes within each group. We calculated the distance between the squared CV of each gene and a running median after excluding genes with a mean expression level lower than ten counts.

To measure transcriptional noise, we measured all possible (d) (based on rank correlation) using the top 500 highly variable genes of each group. We then transformed these correlation measures into distance using the equation below:

$$\text{d}=\sqrt{\frac{\left(1-\text{ρ}\right)}{2}}$$

d = transcriptional noise
ρ = Spearman’s rank correlation coefficient

Cell-cycle stage of the cell groups used for the noise analysis was predicted using a classification algorithm previously described (Scialdone et al., 2015).

## Author Contributions

H.M., I.H.H., and A.S. contributed to design, execution, and interpretation of the project. A.S., I.M., C.M., T.C., and T.V. provided technical guidance and advice. W.D. and J.N. performed embryo dissections and collection. W.D., J.N., J.C.M., and W.R. helped with discussions, interpretation of data, writing the manuscript, and also provided overall guidance and supervision. All authors have contributed with helpful discussions, advice, and recommendations on writing the manuscript.

## Accession Numbers

The accession number for the RNA-seq data reported in this paper is GEO: GSE100597.

## Supplemental Information

Document S1. Figures S1–S5

Table S1. SC3 Gene Clustering by Lineage and List of Genes Used to Define Signaling Pathways, Related to Figures 1 and S1

Table S2. List of Differentially Expressed Genes between the Epiblast and PrE/VE at E4.5, E5.5, and E6.5, Related to Figure 1

Table S3. List of Genes Correlated or Anticorrelated with X Chromosome Expression Levels by Stage, Related to Figure 2

Table S4. List of Late Epiblast and PrE Genes, List of Transcription Factors Identified by EnrichR Analysis, Related to Figure 4

Table S5. List of Differentially Expressed Genes between the AVE and VE at E6.5, Epiblast, and the Primitive Streak, E6.5, Related to Figures 5 and S4

Document S2. Article plus Supplemental Information
