# Supplementary material for: Single-Cell Landscape of Transcriptional Heterogeneity and Cell Fate Decisions during Mouse Early Gastrulation
Source: Cell Rep. 2017 Aug 1;20(5):1215–28. doi: 10.1016/j.celrep.2017.07.009 (PMC5554778; doi:10.1016/j.celrep.2017.07.009)
Supplement: Document S1. Figures S1–S5 [file mmc1.pdf]

Cell Reports, Volume *20*

Supplemental Information

Single-Cell Landscape of Transcriptional Heterogeneity and Cell Fate Decisions during Mouse Early Gastrulation

Hisham Mohammed, Irene Hernando-Herraez, Aurora Savino, Antonio Scialdone, Iain Macaulay, Carla Mulas, Tamir Chandra, Thierry Voet, Wendy Dean, Jennifer Nichols, John C. Marioni, and Wolf Reik

S.1

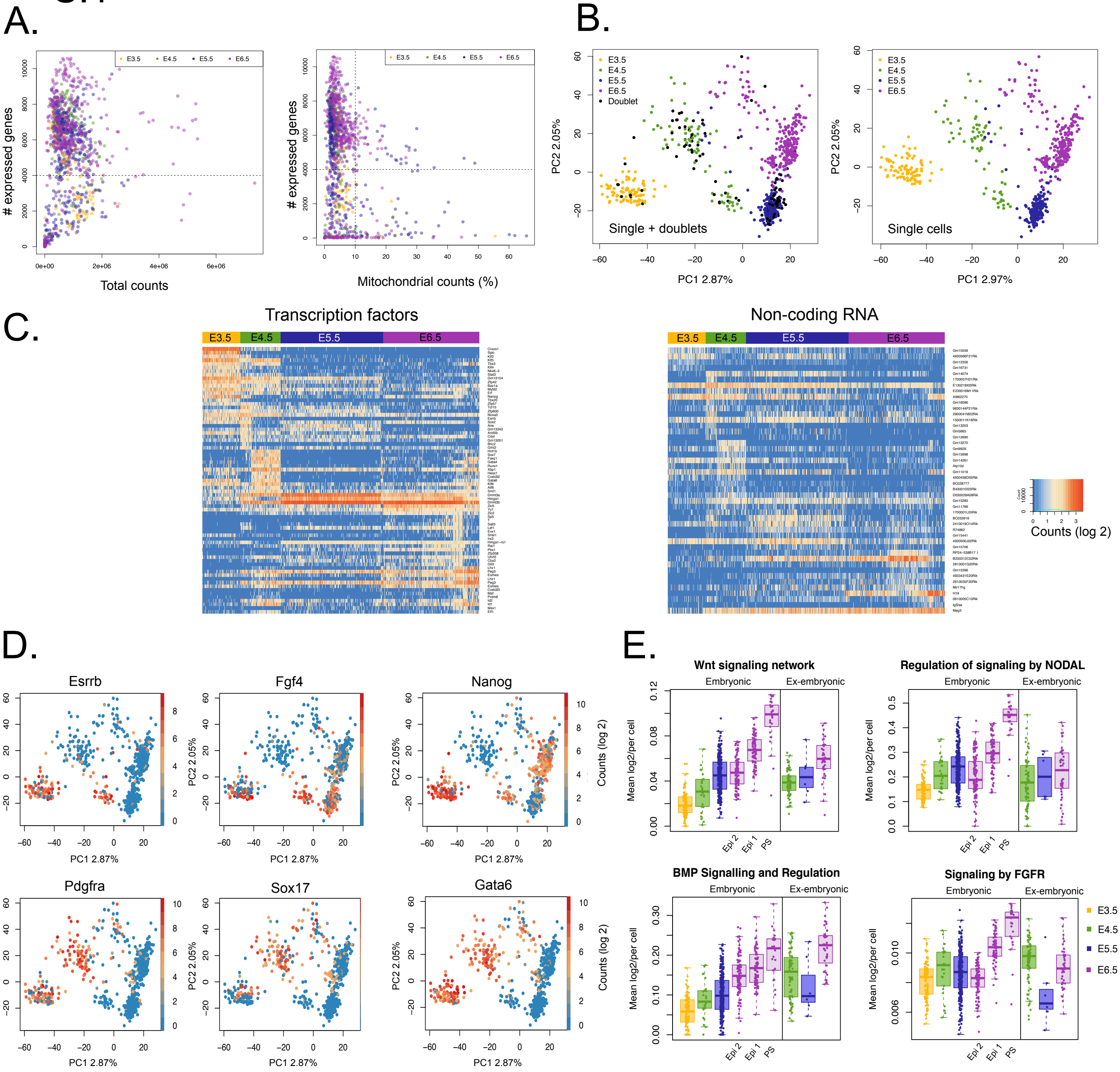

Figure S1 related to Figure 1 (A) Plot representing number of genes expressed in each cell and total expression count (left) or expression from mitochondrial genes for all cells (right). Cells above highlighted threshold (4000 genes, <10% mitochondrial reads) were included in the study. **(B)** PCA plots of cells included in the study to compare PCA showing single cells only or single and double cells. **(C)** PCA plots coloured by the expression levels (log2) of epiblast and endoderm markers. **(D)** Heatmap of transcription factors (left) and non-coding RNA (right) indicating genes specific to developmental and lineage classifications. **(E)** Single cell visualisation of signalling pathways based on SC3 clustering definitions. Each dot represents the mean expression of a pathway (log2) for a single cell.

S.2

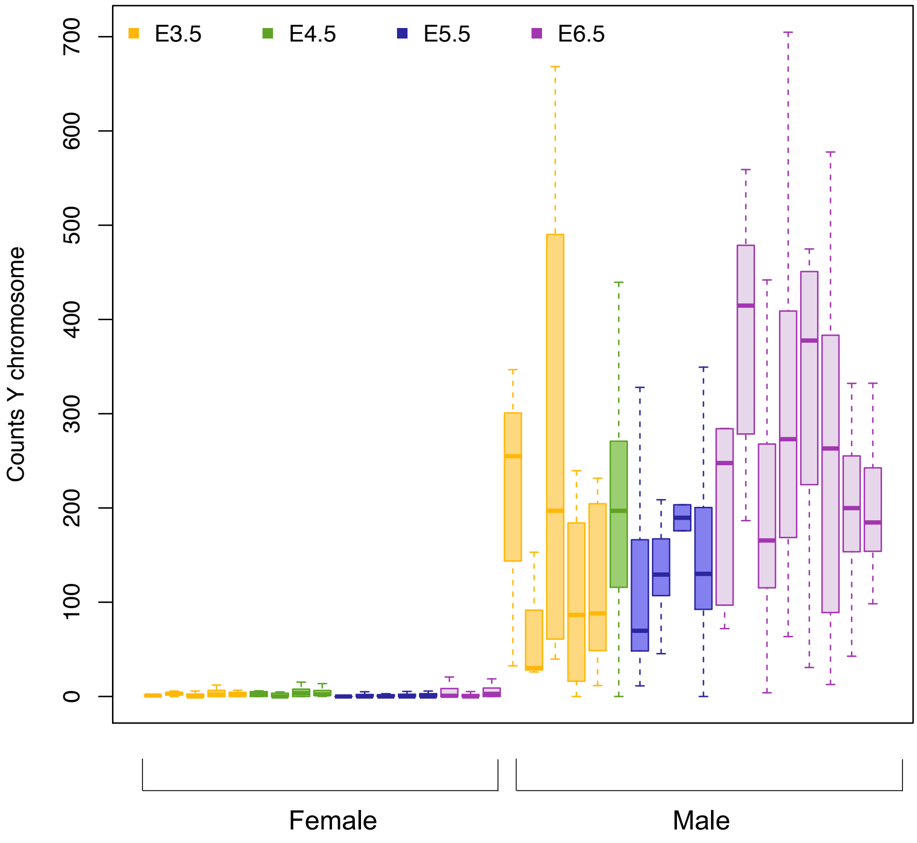

Figure S2 related to Figure 2: Total gene expression counts from the Y-chromosome plotted by embryo.

S.3

A.

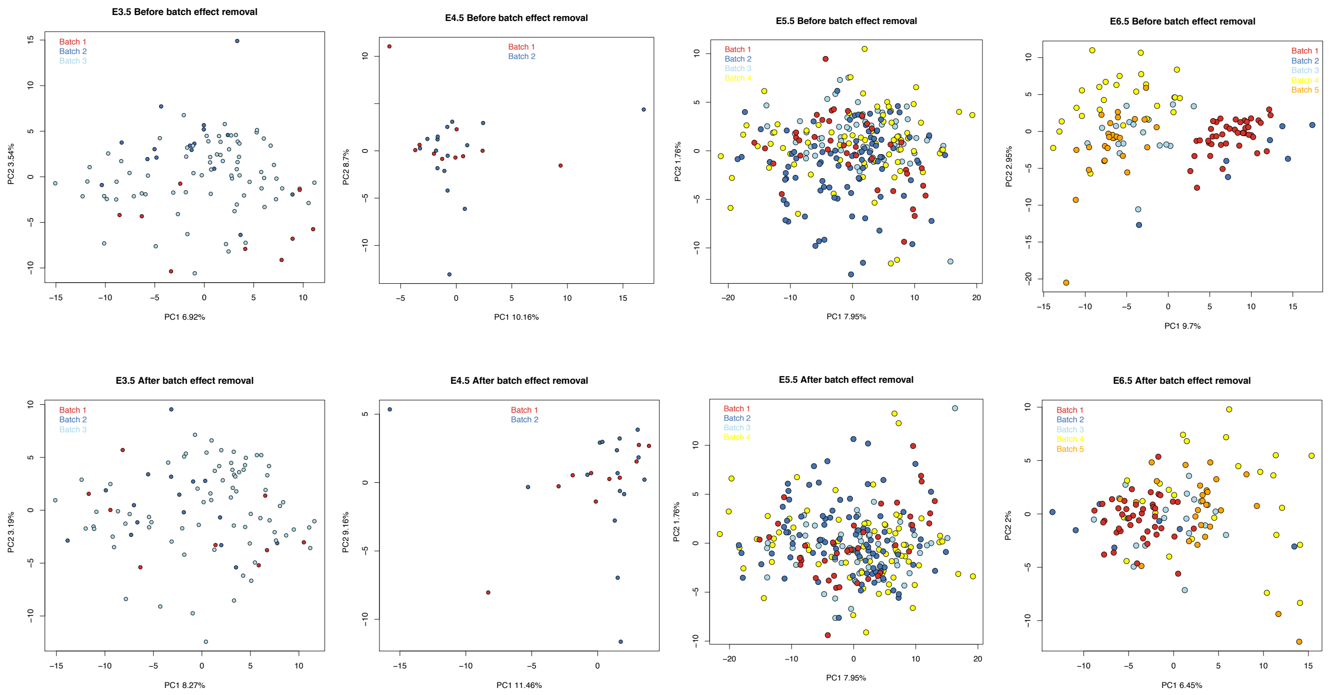

B.

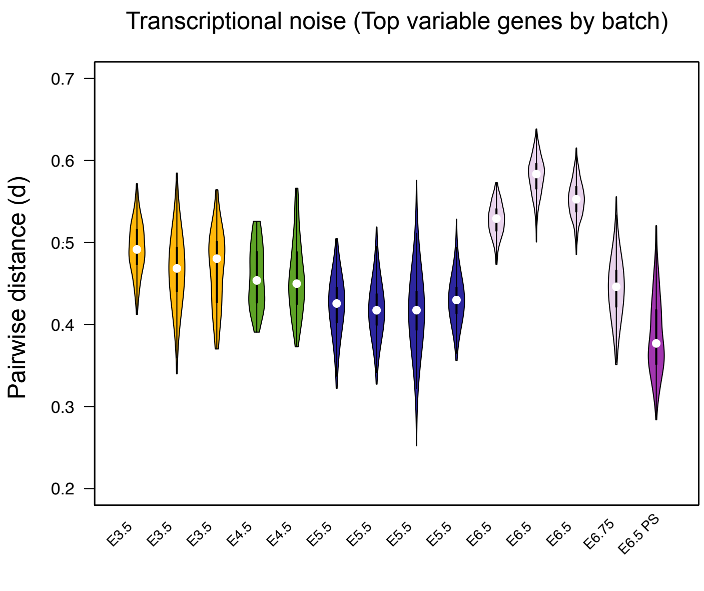

C.

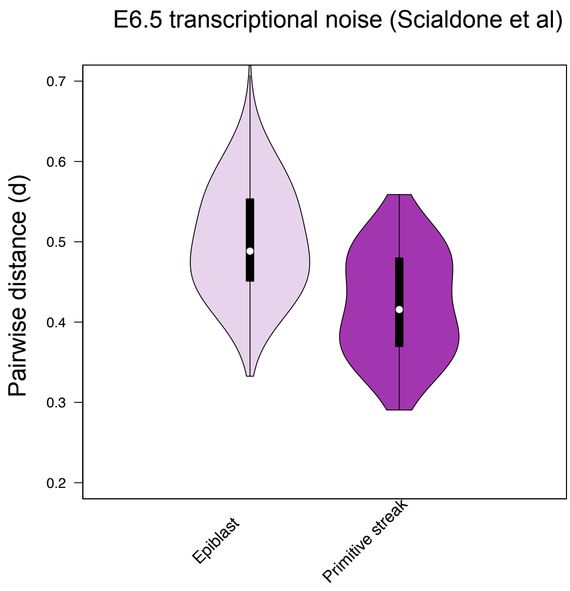

Figure S3 related to Figure 3: **(A)** PCA plot of cells at each stage, coloured by technical batch. Plots show PCA before and after batch correction. **(B)** Plot showing transcriptional noise (d) across all stages separated by technical batch. **(C)** Plot showing transcriptional noise (d) at E6.5 separated by lineage from an independent dataset (Scialdone et al., 2016).

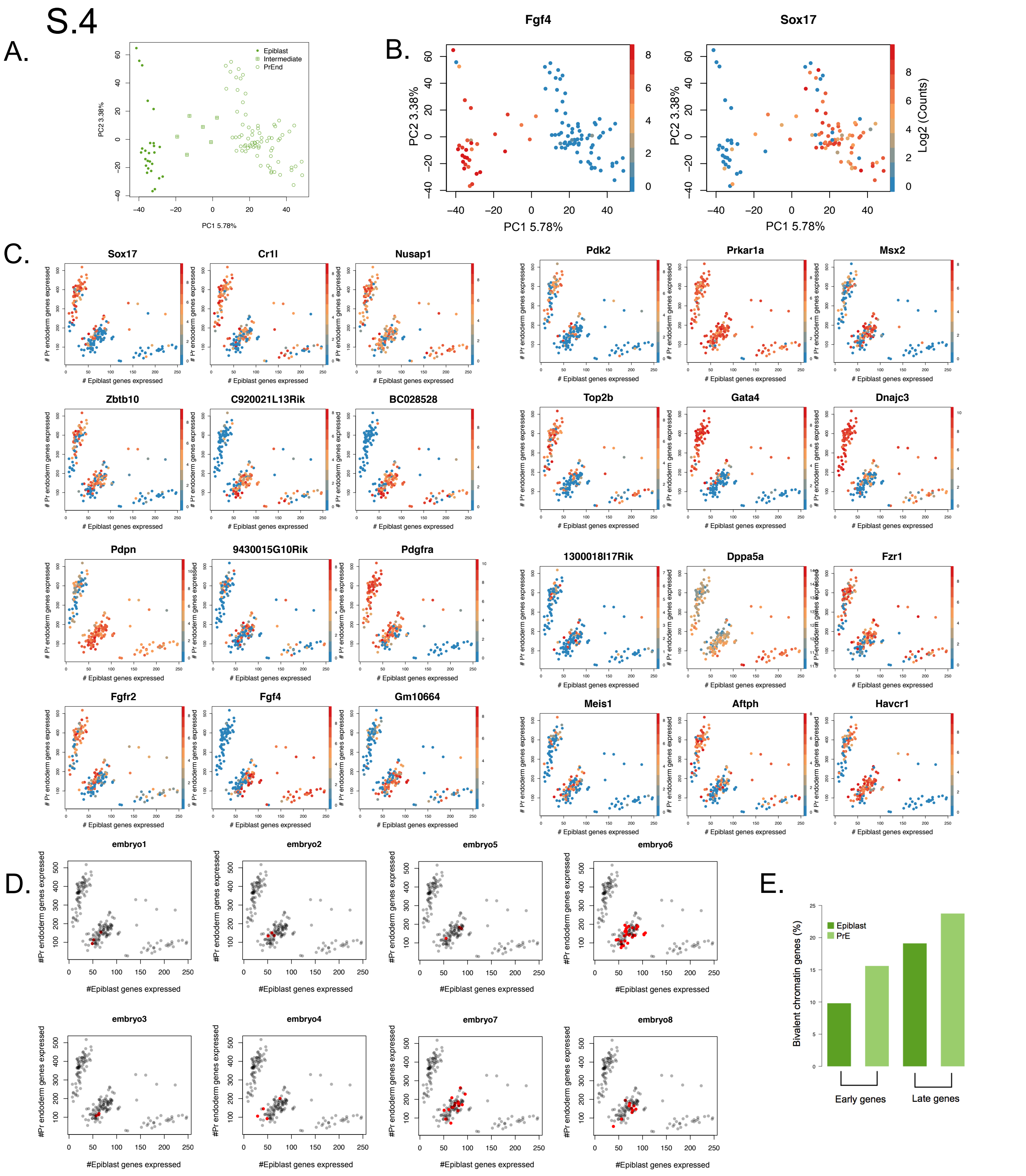

Figure S4 related to Figure 4 (A) PCA plot of E4.5 epiblast, PrE and intermediate cells. **(B)** PCA of E4.5 cells coloured by epiblast (Fgf4) and PrE (Sox17) markers. **(C)** Plots showing number of epiblast and PrE genes expressed in each E3.5 and E4.5 cell. Colour represents expression levels (log2) of the gene. **(D)** Plots showing number of epiblast and PrE genes expressed in each cell coloured by embryo. **(E)** Graph representing percentage of PrE and epiblast genes overlapping with bivalent chromatin regions in ES cells (Rugg-Gunn et al, 2010). Late PrE and epiblast genes show increased enrichment over their early counterparts (P<0.0001).

S.5

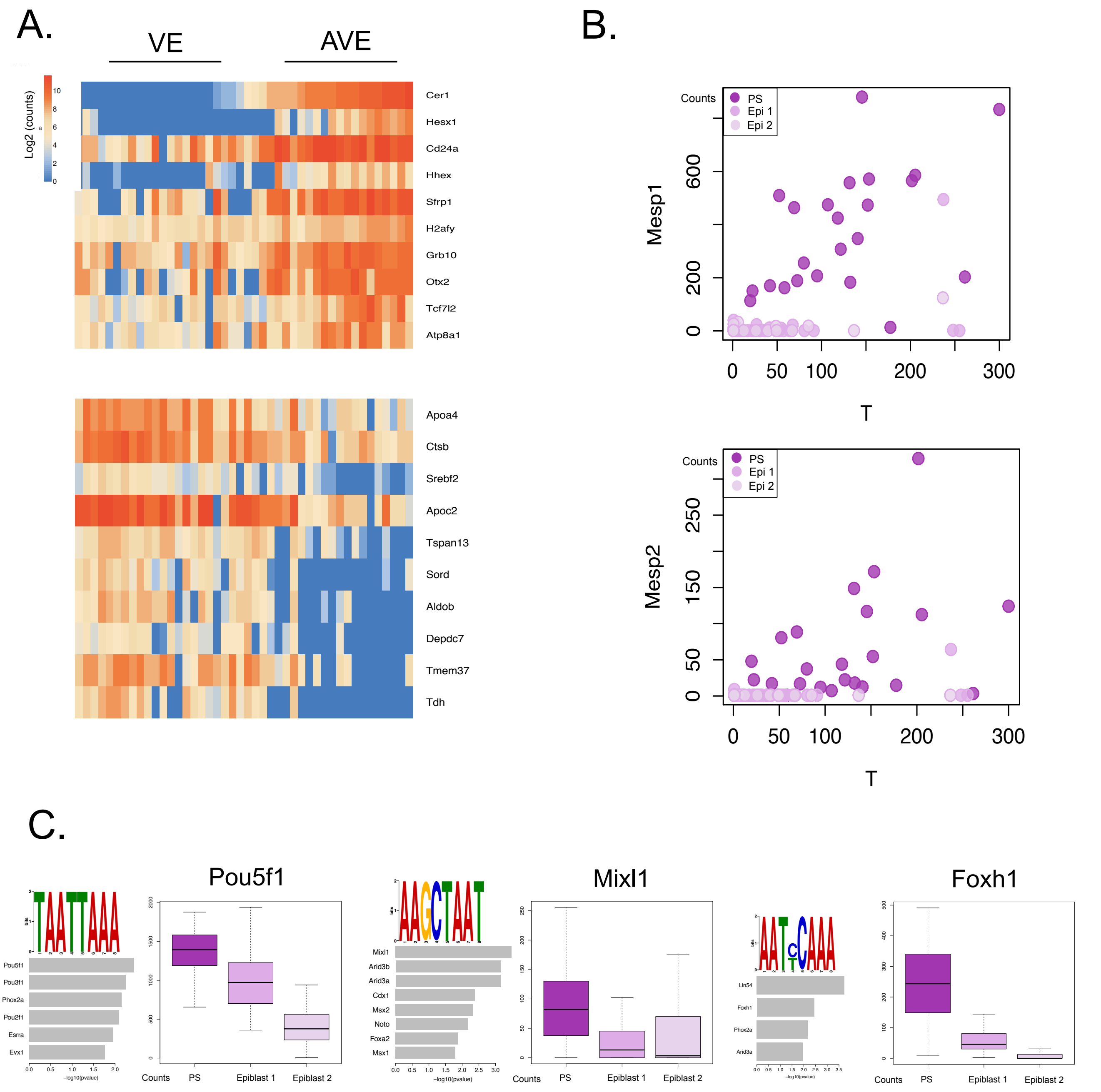

Figure S5 related to Figure 5 (A) Heatmap representing top genes correlating or anti-correlating with Cer1 expression **(B)** Expression levels of primitive streak markers *T*, *Mesp1* and *Mesp2* showing distinct subgroups. **(C)** Top motifs identified in the promoters (500 bp upstream of the TSS) of genes upregulated in the primitive streak.
